# Tissue-Specific Profiles of Free and Bound Phenolics and Their In Vitro Antioxidant and Glycemic-Regulation-Related Activities in Fingered Citron (*Citrus medica* L. var. *sarcodactylis* (Siebold ex Hoola van Nooten) Swingle)

**DOI:** 10.3390/antiox15050564

**Published:** 2026-04-29

**Authors:** Wenkai Guo, Guodong Zhuang, Jin Zhang, Huaming He, Yan Jia, Yongsheng Chen

**Affiliations:** 1 Department of Food Science and Engineering, Jinan University, Guangzhou 510632, China; 2 Beijing Key Laboratory of Plant Resources Research and Development, School of Light Industry Science and Engineering, Beijing Technology and Business University, Beijing 100048, China; hehuaming@btbu.edu.cn; 3 State Key Laboratory of Quality Research in Chinese Medicines, Macau University of Science and Technology, Macau SAR 999078, China

**Keywords:** fingered citron, free and bound phenolics, antioxidant capacity, α-glucosidase inhibition, IR-HepG2 cells, glucose consumption, functional food ingredients

## Abstract

Background/Objectives: Fingered citron (*Citrus medica* L. var. *sarcodactylis* (Siebold ex Hoola van Nooten) Swingle) is rich in phenolic constituents, yet systematic comparisons of free and bound phenolics across tissues and origins remain limited. This study compared the peel, pulp, and blend (whole fruit) of fingered citron from five Chinese regions: Zhejiang (ZJ), Yunnan (YN), Sichuan (SC), Guangdong (GD), and Guangxi (GX). Methods: Phenolic compositions were determined by colorimetric assays and HPLC. Antioxidant activity was assessed by ORAC and PSC, and hypoglycemic-related activity by α-glucosidase and α-amylase inhibition and glucose consumption in an insulin-resistant HepG2 (IR-HepG2) cell model. Results: Phenolic distribution followed the order peel > blend > pulp, and free > bound. HPLC identified 11 free and 5 bound phenolics, predominantly hesperidin, quercetin, and 5,7-dimethoxycoumarin. GX peel exhibited the highest free phenolic content (106.34 ± 0.23 mg GAE/100 g FW) and superior ORAC (30.56 ± 0.50 μmol TE/g FW), strongly correlating with total phenolics (*r* = 0.98, *p* < 0.01). Free phenolics showed stronger α-glucosidase and α-amylase inhibition, whereas bound phenolics produced higher glucose consumption in the IR-HepG2 cell model. The GX blend bound fraction showed the highest glucose consumption (5.48 ± 0.98 mmol/L). Conclusions: Under fresh-weight-based conditions, phenolic composition and in vitro bioactivities differed by fruit part, region, and phenolic fraction. Peel, especially GX peel, tended to show higher phenolic levels and stronger antioxidant-related performance, whereas the GX blend bound fraction showed the highest glucose-consumption-promoting activity.

## 1. Introduction

Fingered citron (*Citrus medica* L. var. *sarcodactylis* (Siebold ex Hoola van Nooten) Swingle), commonly known as “Fo-Shou” or “Buddha’s hand”, is a unique species within the Citrus genus of the Rutaceae family [1]. Renowned for its unique fruit morphology and broad utility, fingered citron has high economic value and promising applications in food, cosmetic, and health-related products [2].

Beyond its commercial applications, fingered citron is increasingly recognized for its abundant bioactive components, particularly phenolic compounds, which have demonstrated diverse health-promoting properties [3]. These phenolics exhibit potent antioxidant, anti-inflammatory, and lipid-lowering activities, making fingered citron a promising candidate for functional food and nutraceutical applications, particularly in the management of metabolic disorders such as diabetes [4,5].

Diabetes is a multifactorial metabolic disorder characterized by chronic hyperglycemia and oxidative stress, leading to severe complications such as cardiovascular disease and neuropathy [6]. Insulin resistance, a hallmark of diabetes, results in impaired glucose consumption, elevated blood glucose levels, and excessive production of reactive oxygen species (ROS), which further exacerbate oxidative stress and disease progression [7]. While conventional anti-diabetic therapies, such as insulin injections and synthetic glucose-lowering drugs, are effective, their long-term use is often associated with high costs and undesirable side effects [8]. This has led to a growing interest in plant-derived bioactive compounds as safer, cost-effective, and multifunctional alternatives [9].

Previous studies have reported that fingered citron extracts exhibit anti-diabetic activities, particularly through inhibitory effects on key carbohydrate-hydrolyzing enzymes such as α-glucosidase and α-amylase [10]. These activities have been loosely associated with the presence of phenolic constituents in fingered citron. However, despite promising findings, most reports assess phenolic composition and bioactivity largely in isolation, with limited systematic comparisons across fruit tissues and geographical origins, which weakens the evidence for composition–activity relationships. This is particularly relevant because phenolic accumulation in citrus fruits is strongly influenced by geographical origin and associated agroclimatic factors (e.g., temperature, sunlight), which may lead to substantial variations in phytochemical profiles and bioactivities [11]. Therefore, region-resolved evaluation is important for the standardization and value-added utilization of fingered citron resources.

To address these gaps, this study systematically compared the phytochemical profiles and in vitro bioactivities of fingered citron fruits collected from five representative production regions in China. Specifically, the objectives were to (1) quantify total phenolics, flavonoids, flavonols, and individual phenolic compounds in different tissues and phenolic fractions; (2) evaluate the antioxidant capacities of these extracts using ORAC and PSC assays; and (3) assess their glycemic-regulation-related potential through digestive enzyme inhibition and glucose consumption in an insulin-resistant HepG2 (IR-HepG2) cell model. By integrating compositional and functional data, this work provides comparative evidence for the value-added utilization of different fingered citron tissues and highlights differences between free and bound phenolic fractions.

## 2. Materials and Methods

### 2.1. Materials

Sodium hydroxide (AR) and sodium carbonate (AR) were purchased from Tianjin Damao Chemical Reagent Factory, Tianjin, China. Gallic acid, 7-hydroxycoumarin, and catechin (AR) were obtained from Shanghai Aladdin Biochemical Technology Co., Ltd., Shanghai, China. Isoscopoletin (HPLC grade) was obtained from Sichuan Vikqi Biotechnology Co., Ltd., Chengdu, China. Ethanol (AR) was purchased from Guangdong Guanghua Sci-Tech Co., Ltd., Shantou, China. Acetic acid (HPLC grade) was obtained from Tianjin Kemeiou Chemical Reagent Co., Ltd., Tianjin, China. Trolox (AR) was purchased from Saen Chemical Technology (Shanghai) Co., Ltd., Shanghai, China. A glucose oxidase assay kit (96 T) was purchased from Nanjing Jiancheng Bioengineering Institute, Nanjing, China. DMEM high-glucose medium, penicillin-streptomycin solution, and trypsin were obtained from Gibco (Thermo Fisher Scientific, Waltham, MA, USA). Fetal bovine serum was purchased from Zhejiang Tianhang Biotechnology Co., Ltd., Deqing, China. HepG2 cells were donated by Prof. Wenzhen Liao from Southern Medical University, Guangzhou, China. Information on the five types of fingered citron is summarized in Table 1. Fresh fingered citron samples were used throughout the extraction procedures, and all quantitative results in this study were expressed on a fresh-weight (FW) basis.

### 2.2. Extraction of Free Phenolics

Fresh fingered citron fruits were used throughout the study. Before extraction, the fruits were manually peeled. The peel fraction consisted of the manually separated outer peel, the pulp fraction was obtained from the peeled fruit, and the blend fraction represented the whole fruit sample processed without tissue separation. Free phenolics were extracted using a modified procedure based on Guo [12]. Fresh fingered citron samples (60 g; cut into approximately 0.5 × 0.5 × 0.5 cm pieces) were first mixed with n-hexane at a ratio of 1 g:10 mL and stirred magnetically for 10 min. This pretreatment step was repeated three times. After vacuum filtration, the solid residue was collected and soaked in 70% aqueous ethanol at 4 °C for 24 h. The mixture was then homogenized in an ice bath at 14,000 rpm for 3 min, and this homogenization step was repeated three times. After filtration, the filtrate was concentrated under reduced pressure at 40 °C, adjusted to 10 mL with deionized water, aliquoted, and stored at −40 °C until analysis. The solid residue remaining after free phenolic extraction was used for subsequent extraction of bound phenolics. For all free phenolic extracts, the concentrates were adjusted to a fixed final volume of 10 mL for subsequent comparative chemical and bioactivity analyses.

### 2.3. Extraction of Bound Phenolics

Bound phenolics were extracted using a modified alkaline hydrolysis procedure adapted from the method reported by Alu’datt [13] and the extraction procedure described by Chen [14]. The solid residue remaining after the free phenolic extraction was flushed with nitrogen gas, tightly sealed, and digested with 20 mL of 4 M NaOH. The mixture was then agitated at 60 rpm at room temperature for 90 min. Following the alkaline hydrolysis, the mixture was acidified to pH 2.0 using HCl to liberate the bound phenolic compounds. The acidified solution was transferred to a separatory funnel and extracted with ethyl acetate at a ratio of 1:1 (*v*/*v*). The upper ethyl acetate layer was collected, and this liquid–liquid extraction was repeated six times. The combined ethyl acetate fractions were concentrated under reduced pressure at 40 °C, adjusted to a final volume of 10 mL with deionized water, aliquoted, and stored at −40 °C until analysis. This alkaline hydrolysis condition was selected with reference to previously reported alkaline extraction procedures and was applied uniformly to all samples for comparative extraction of bound phenolics. For all bound-phenolic extracts, the concentrates were adjusted to a fixed final volume of 10 mL for subsequent comparative chemical and bioactivity analyses.

### 2.4. Determination of the Phenolic Content

The phenolic content was determined using the Folin–Ciocalteu colorimetric method [15]. A standard curve was prepared using gallic acid (0–600 µg/mL). Briefly, 100 µL of extract, 400 µL of deionized water, and 100 µL of Folin–Ciocalteu reagent were mixed. After a 6 min reaction, 1 mL of 7% Na_2_CO_3_ was added, followed by 0.8 mL of deionized water. The mixture was incubated at room temperature for 90 min. The absorbance was measured at 760 nm using a microplate reader. Phenolic content was calculated as mg gallic acid equivalents (GAE)/100 g FW based on the standard curve.

### 2.5. Determination of the Flavonoid Content

The flavonoid content was quantified using the aluminum chloride colorimetric method [16], with catechin as a reference compound. A standard curve was prepared using catechin standard solutions (0–100 µg/mL). Briefly, 1 mL of extract or catechin standard solution was mixed with 4 mL of deionized water and 0.3 mL of sodium nitrite. After 5 min of reaction, 0.3 mL of 10% AlCl_3_ was added, followed by 2 mL of 4% NaOH after 6 min. The absorbance was measured at 510 nm, and the flavonoid content was expressed as mg catechin equivalents (CE)/100 g FW.

### 2.6. Determination of the Flavonol Content

The flavonol content was determined using the aluminum chloride-sodium acetate colorimetric method [17], with rutin as the reference compound. A standard curve was prepared using rutin standard solutions (0–100 µg/mL). Briefly, 0.2 mL of the sample or rutin standard solution was mixed with 0.2 mL of 20 mg/mL AlCl_3_ and 0.6 mL of 50 mg/mL sodium acetate. After a 2.5 h incubation, the optical density (OD) was measured at 440 nm. Flavonol content was reported as mg rutin equivalents (RE)/100 g FW.

### 2.7. ORAC Assay

The ORAC was determined as described by Chen [14]. In a 96-well plate, 100 µL of PBS, Trolox, or fingered citron extract was added. After 10 min of incubation at 37 °C, 100 µL of fluorescein was added, followed by a 20 min incubation. AAPH (90 µL of 200 mM) was added, and fluorescence was measured at 485/538 nm every 4.5 min for 35 cycles. The area under the curve (AUC) was calculated, and ORAC values were expressed as µmol Trolox equivalents (TE) per gram of fresh weight (µmol TE/g FW).

### 2.8. PSC Assay

The peroxyl radical scavenging activity was evaluated according to the method described by Adom [18]. In a black 96-well plate, 100 µL of either vitamin C standard or fingered citron extract was mixed with 100 µL of 13.26 µM DCFH solution and 50 µL of 200 mM AAPH solution. The plate was incubated in a microplate reader at 37 °C, and fluorescence was measured every 2 min over a 40 min period at 485 nm (excitation) and 538 nm (emission). The assay was performed in triplicate, and the average fluorescence values were used to calculate the area under the curve (AUC). Antioxidant activity was calculated according to Equation (1):

(1) $$\mathrm{PSC}\;\mathrm{unit}\;=\;1-\left(\mathrm{SA}/\mathrm{CA}\right)$$

In this equation, SA represents the area under the curve (AUC) for the sample or standard dilution, while CA represents the AUC for the control reaction group in which only buffer solution was used. The standard and extract inhibited the oxidation of DCFH, thereby lowering SA and increasing the PSC value. Results were expressed as micromoles of vitamin C equivalents per gram of sample (μmol VCE/g FW).

### 2.9. HPLC Analysis

HPLC analysis of fingered citron extracts was performed using a modified procedure adapted from Zhang [19]. Separation was achieved on an Agilent ZORBAX SB-C18 column (250 mm × 4.6 mm, 5 μm). The mobile phases consisted of acetonitrile (A) and 0.1% aqueous acetic acid (B), with binary gradient elution as follows: 0–40 min, 5–25% A; 40–45 min, 25–35% A; 45–50 min, 35–50% A; 50–60 min, 50–25% A; 60–61 min, 25–5% A; and 61–70 min, 5% A. The flow rate was 1.0 mL/min, the column temperature was 30 °C, and the injection volume was 10 μL. Prior to HPLC analysis, the reconstituted extracts were filtered through a 0.45 μm membrane and diluted appropriately, when necessary, to ensure that the peak areas of the target compounds fell within the linear ranges of the corresponding standard curves. Quantification was performed using the corresponding calibration equations (Table 2), and the final contents were corrected according to the respective dilution factors. Chromatograms were recorded using one detector with two monitored channels at 283 and 320 nm. These two wavelengths were selected to improve the detection coverage of the main target phenolic acids, flavonoids, and coumarin derivatives in fingered citron extracts. The present HPLC analysis was applied as a targeted comparative profiling method under unified chromatographic conditions for all samples.

The 11 reference standards used for peak identification and quantification were gallic acid, chlorogenic acid, caffeic acid, isoscopoletin, ferulic acid, rutin, hesperidin, neohesperidin, quercetin, 7-hydroxycoumarin, and 5,7-dimethoxycoumarin. These target compounds were selected as a targeted representative panel to cover the major classes relevant to the present free and bound phenolic fractions, namely phenolic acids, flavonoids, and coumarin derivatives, based on previous reports on fingered citron and related citrus materials together with the availability of authenticated reference standards [20,21]. The calibration equations and coefficients of determination (R^2^) of all 11 reference standards are summarized in Table 2.

### 2.10. α-Glucosidase Inhibition Assay

The α-glucosidase inhibition was measured using a method adapted from Chen [22]. In a 96-well plate, 110 μL of PBS, 20 μL of α-glucosidase (0.5 U/mL), and 10 μL of either the standard or fingered citron extract were incubated at 37 °C for 15 min. Then, 20 μL of 2.5 mM p-nitrophenyl-α-D-glucopyranoside (pNPG) was added, and the mixture was incubated for an additional 15 min at 37 °C. The reaction was terminated by adding 80 μL of 0.2 M Na_2_CO_3_, and the optical density (OD) was measured at 405 nm. α-Glucosidase inhibition was calculated according to Equation (2). The inhibition percentage was subsequently converted to acarbose equivalents (AE) using an acarbose calibration curve and expressed as mg AE/100 g FW.

(2) $$\alpha \text{-Glucosidase}\;\mathrm{inhibition}\;(\%)=[1-(A_{s}-A_{sb})/(A_{c}-A_{b})]\;\times \;100$$

where A_s_ is the absorbance of the reaction mixture containing extract, α-glucosidase, and pNPG; A_sb_ is the absorbance of the corresponding sample blank in which α-glucosidase was replaced with PBS; A_c_ is the absorbance of the control reaction containing α-glucosidase and pNPG but no extract; and A_b_ is the absorbance of the blank reaction in which α-glucosidase was replaced with PBS and no extract was added.

### 2.11. α-Amylase Inhibition Assay

The α-amylase inhibition was determined as per Oluwagunwa [23]. Fingered citron extract and acarbose were dissolved in PBS (pH 6.8). α-Amylase (20 U/mL) and soluble starch substrate (10 g/L) were prepared in PBS and preheated water, respectively. The DNS color reagent was prepared by dissolving 3.25 g of 3,5-dinitrosalicylic acid in water, followed by the addition of sodium hydroxide and glycerol, and then diluted to a final volume of 500 mL. In 1.5 mL centrifuge tubes, 40 μL of α-amylase and 20 μL of either acarbose or fingered citron extract were mixed and incubated at 37 °C for 15 min. After adding 40 μL of starch substrate, the mixture was incubated for an additional 15 min at 37 °C. The reaction was terminated by adding 200 μL of ethanol and 150 μL of DNS reagent, followed by boiling for 5 min. The samples were cooled and diluted with 200 μL of deionized water, and the optical density (OD) was measured at 540 nm. α-Amylase inhibition was calculated according to Equation (3). The inhibition percentage was subsequently converted to acarbose equivalents (AE) using an acarbose calibration curve and expressed as mg AE/100 g FW.

(3) $$\alpha \text{-Amylase}\;\mathrm{inhibition}\;(\%)=[1-(A_{s}-A_{sb})/(A_{c}-A_{b})]\;\times \;100$$

where A_s_ is the absorbance of the reaction mixture containing extract, α-amylase, and starch substrate; A_sb_ is the absorbance of the corresponding sample blank in which α-amylase was replaced with PBS; A_c_ is the absorbance of the control reaction containing α-amylase and starch substrate but no extract; and A_b_ is the absorbance of the blank reaction in which α-amylase was replaced with PBS and no extract was added.

### 2.12. Cytotoxicity Assay

The cytotoxicity was assessed using the methylene blue staining method as described by Felice [24]. HepG2 cells (2.0 × 10^4^/well) were seeded in a 96-well plate and incubated at 37 °C for 24 h under 5% CO_2_. After washing with PBS, varying concentrations of fingered citron phenolic extract were added, and incubation continued for another 24 h. Cells were washed with PBS, stained with 50 µL methylene blue solution, and incubated at 37 °C for 1 h, followed by another PBS wash. After drying, 100 µL of elution solution (49% PBS, 50% ethanol, 1% acetic acid) was added and shaken for 20 min to dissolve the dye. Optical density (OD) was measured at 570 nm, and cytotoxicity was calculated using Equation (4):

(4) $$C\mathrm{ytotoxicity}(\%)\;=\;[(A_{c}-A_{s})/(A_{c}-A_{0})]\;\times \;100$$

A_c_, A_s_ and A_0_ denote the optical densities of the control group, sample group, and blank group, respectively. Cytotoxicity (%) reflects the reduction in OD of the sample group compared to the control.

### 2.13. HepG2 Cell Proliferation Assay

Cell proliferation was determined according to the method described by Chen [25]. HepG2 cells (1.0 × 10^4^/well) were seeded in a 96-well plate and incubated for 4 h under 5% CO_2_ to allow adhesion. Cells were treated with various concentrations of fingered citron phenolic extract for 48 h, washed with PBS, and stained with 50 µL methylene blue for 1 h. After drying, elution solution was added, and the plates were shaken for 20 min to dissolve the dye. Optical density (OD) was measured at 570 nm, and cell proliferation activity was calculated using Equation (5):

(5) $$C\mathrm{ell}\;\mathrm{proliferation}(\%)\;=\;[(A_{s}-A_{0})/(A_{c}-A_{0})]\;\times \;100$$

A_c_, A_s_ and A_0_ denote the OD values of the control, sample, and blank groups, respectively. The reduction in OD of the sample group relative to the control reflects its effect on cell proliferation.

### 2.14. Determination of Glucose Consumption in IR-HepG2 Cells

The glucose consumption in insulin-resistant HepG2 cells was determined according to a previously reported HepG2 glucose-consumption assay with minor modifications [22]. Logarithmic-phase HepG2 cells (1.0 × 10^4^/well) were seeded in a 96-well plate and incubated at 37 °C for 12 h. After washing with serum-free DMEM high-glucose medium, cells were cultured in DMEM containing 0.5 μM insulin for 24 h to induce insulin resistance. The medium was then replaced, and cells were treated with various concentrations of fingered citron phenolic extract for another 24 h. A positive control (25 mM metformin) and a blank group (DMEM medium) were included. Glucose consumption in IR-HepG2 cells was determined using a glucose oxidase assay kit (Nanjing Jiancheng Bioengineering Institute) following the manufacturer’s instructions.

### 2.15. Data Analysis

Experimental data are presented as mean ± standard deviation. All experiments were performed in triplicate, and statistical analysis was conducted using Origin 2021 (OriginLab Corporation, Northampton, MA, USA) and SPSS 26.0 (IBM Corp., Armonk, NY, USA). Significant differences among groups were evaluated by one-way analysis of variance (ANOVA) followed by Duncan’s multiple range test. Pearson correlation analysis was used to evaluate the relationships between phenolic composition and bioactivities. Differences were considered statistically significant at *p* < 0.05.

## 3. Results

### 3.1. Phenolic Content

**Figure 1:**
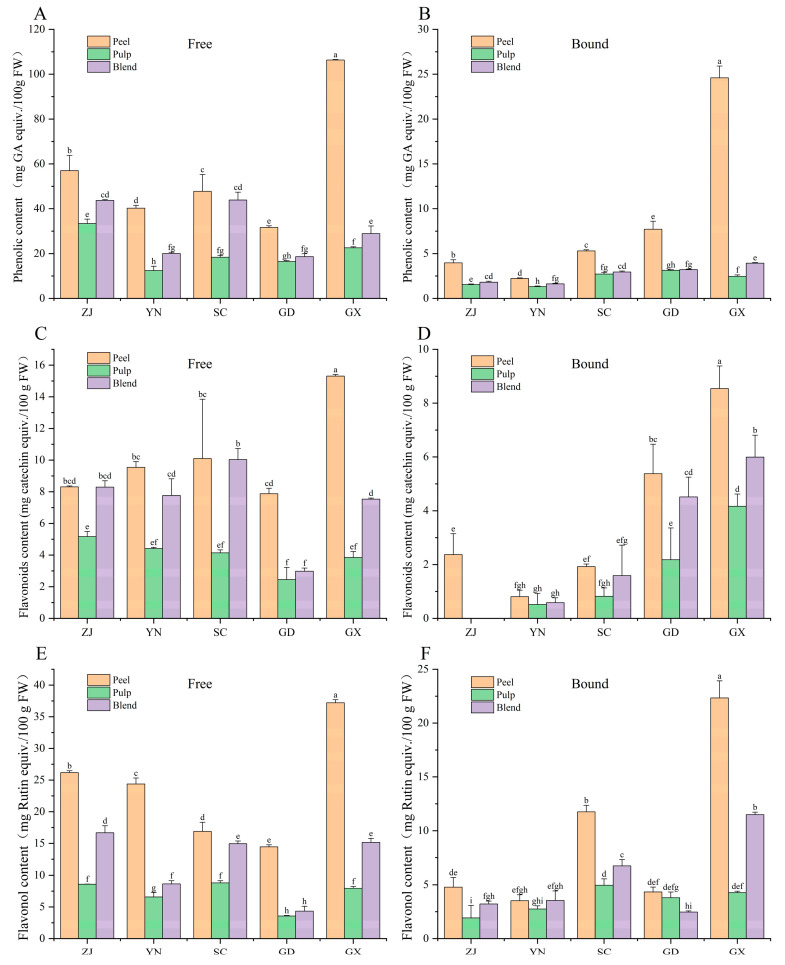
Phenolic, flavonoid, and flavonol contents in free (**A**,**C**,**E**) and bound (**B**,**D**,**F**) phenolic extracts from fingered citron samples collected from five regions. Different lowercase letters indicate significant differences among groups (*p* < 0.05) according to one-way ANOVA followed by Duncan’s multiple range test. Regions: Zhejiang (ZJ), Yunnan (YN), Sichuan (SC), Guangdong (GD), and Guangxi (GX).

The phenolic content (PC) exhibited significant regional and tissue-specific differences (*p* < 0.05). The highest PC was observed in the peel extract from GX (106.34 ± 0.23 mg GAE/100 g FW), which significantly exceeded the second-highest value (*p* < 0.05), ZJ peel (56.99 ± 6.76 mg GAE/100 g FW), by about 85% (Figure 1A). In contrast, the PC of YN pulp was the lowest (12.41 ± 1.94 mg GAE/100 g FW). Peels consistently contained higher levels of phenolic compounds than pulp or blend samples, highlighting the peel’s key role in accumulating bioactive compounds. Following a similar trend in phenolic compounds, the GX peel again showed the highest content at 24.59 ± 1.31 mg GAE/100 g FW, while the YN pulp had the lowest content at 1.31 ± 0.06 mg GAE/100 g FW (Figure 1B). Notably, flavonoid content was not detected in the bound phenolic extracts from ZJ pulp and blends, possibly due to the low concentration of flavonoids or inefficient binding with cell wall components in these tissues. Overall, the PC distribution for all varieties followed the pattern: peel > blend > pulp. These results align with previous studies [26], which identified citrus peels as the key storage sites for bioactive compounds, such as hesperidin and rutin, due to their localized accumulation during fruit development. This further underscores the peel’s importance in storing bioactive phenolic compounds.

### 3.2. Flavonoid and Flavonol Content

The flavonoid content (FC), expressed in mg CE/100 g FW, showed significant differences between varieties and plant parts (Figure 1C,D). The free phenolic extract from GX peel exhibited the highest FC (15.31 ± 0.11 mg CE/100 g FW), which was 50% higher than that of SC peel (10.10 ± 3.75 mg CE/100 g FW) and six times higher than that of GD pulp (2.45 ± 0.77 mg CE/100 g FW). The bound flavonoid content was also the highest in GX peel (8.54 ± 0.84 mg CE/100 g FW). Flavonoids were undetectable in the bound phenolic extracts of ZJ pulp and blend. Overall, the FC distribution for all varieties followed the pattern: peel > blend > pulp. Similarly, flavonol content (measured in mg RE/100 g FW) also followed the same distribution trend. GX peel had the highest free flavonol content at 37.20 ± 0.84 mg RE/100 g FW, the highest among all tested samples (Figure 1E). The bound flavonol content in GX peel was also the highest, reaching 22.33 ± 1.93 mg RE/100 g FW (Figure 1F). These results confirm that citrus peels are rich in flavonoids and flavonols, highlighting their potential for industrial applications.

### 3.3. Phenolic Compound Profiles

**Figure 2:**
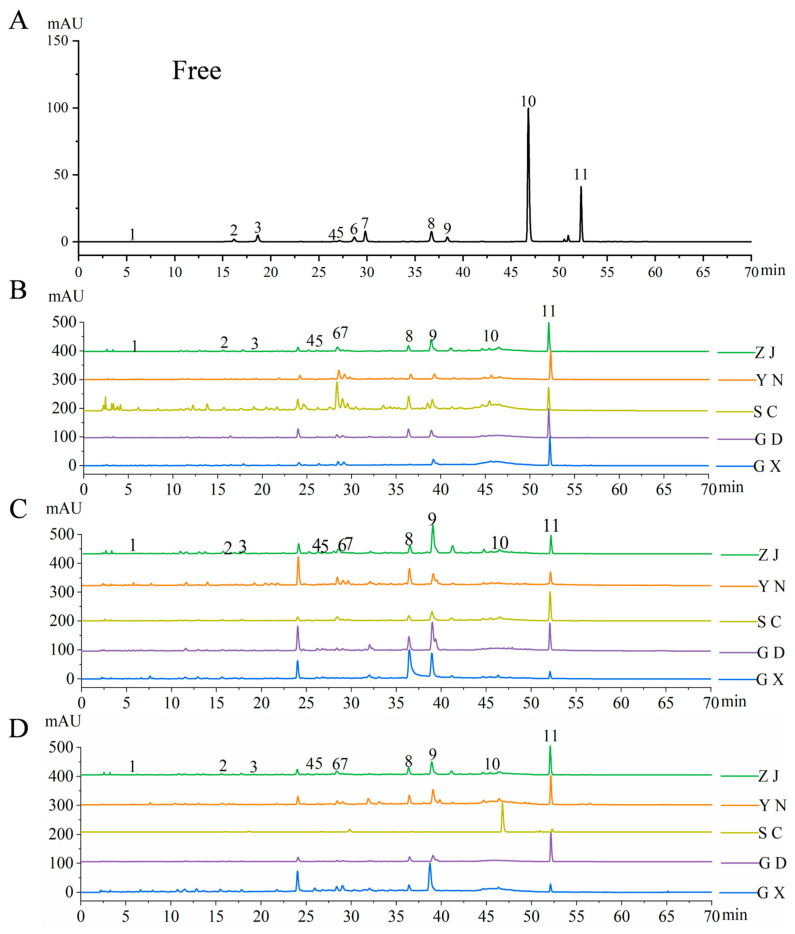
HPLC chromatograms of free phenolics from fingered citron samples. (**A**) Standard mixture of free phenolic compounds; (**B**) Peel; (**C**) Pulp; (**D**) Blend. Identified compounds are as follows: 1—Gallic acid, 2—Chlorogenic acid, 3—Caffeic acid, 4—Isoscopoletin, 5—7-Hydroxycoumarin, 6—Ferulic acid, 7—Rutin, 8—Hesperidin, 9—Neohesperidin, 10—Quercetin, and 11—5,7-Dimethoxycoumarin. Region codes as in Figure 1.

**Figure 3:**
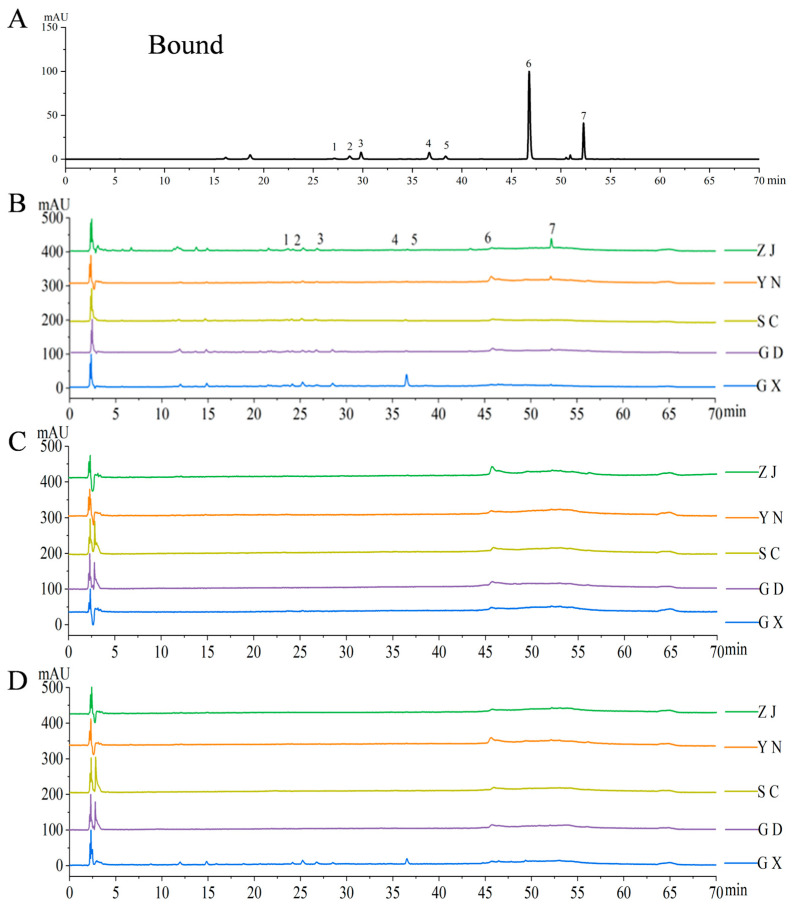
HPLC chromatograms of bound phenolics from fingered citron samples. (**A**) Standard mixture of bound phenolic compounds; (**B**) peel; (**C**) pulp; (**D**) blend. Identified compounds are as follows: 1—7-Hydroxycoumarin, 2—Ferulic acid, 3—Hesperidin, 4—Quercetin, and 5—5,7-Dimethoxycoumarin. Region codes as in Figure 1.

HPLC analysis identified 11 free monomeric phenolic compounds and 5 bound monomeric phenolic compounds in the fingered citron samples, with their representative chromatograms illustrated in Figure 2 and Figure 3. The specific types and concentrations of these monomeric phenolics across different extracts are summarized in Table 3. The results showed that the diversity of bound phenolic compounds was lower, and their concentrations were significantly lower than those of free phenolics. The bound phenolic extracts primarily consisted of a limited number of phenolic acids and flavonoids, including 7-hydroxycoumarin, ferulic acid, hesperidin, and 5,7-dimethoxycoumarin, with hesperidin being the most predominant (1067.13 ± 37.00 μg/g). Among the identified flavonoids, rutin was classified as a flavonol glycoside, whereas hesperidin and neohesperidin were classified as flavanone glycosides. These observed differences in the composition of free and bound phenolics across various samples may be attributed to the intrinsic properties of the raw materials or the potential degradation of specific compounds, such as chlorogenic acid, under alkaline hydrolysis conditions. Additionally, caffeic acid was exclusively detected in the SC and ZJ samples, indicating that geographical origin significantly impacts the specific phenolic profile of fingered citron.

### 3.4. Antioxidant Activity

**Figure 4:**
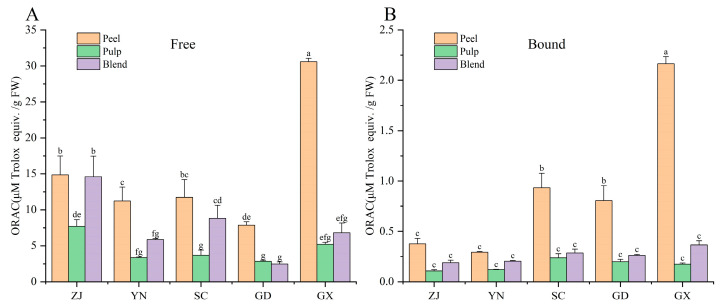
ORAC values of free (**A**) and bound (**B**) extracts from five fingered citron varieties. Different letters indicate significant differences (*p* < 0.05). Region codes as in Figure 1.

The antioxidant activity of fingered citron extracts was evaluated using ORAC and PSC assays. Significant differences in ORAC values were observed across different parts of fingered citron, and the antioxidant activity ranked as follows: peel > blend > pulp. The free phenolic extract from GX peel exhibited the highest ORAC value (30.56 ± 0.50 μmol TE/g FW), significantly outperforming the other samples (*p* < 0.05) (Figure 4A). In the bound phenolic extracts, GX peel also demonstrated the strongest antioxidant activity, with an ORAC value of 2.16 ± 0.07 μmol TE/g FW (Figure 4B). A strong positive correlation was observed between total phenolic content and ORAC values (*r* = 0.98), while PSC also showed a positive correlation with total phenolic content (*r* = 0.77) in line with Suleria [27], who reported a similar correlation between citrus phenolic compounds and antioxidant activity. In the PSC assay, the free phenolic extract from ZJ peel exhibited the highest peroxide radical scavenging activity (0.22 ± 0.01 μmol VCE/g FW), significantly higher than other fingered citron samples (*p* < 0.05). YN peel followed with a PSC value of 0.19 ± 0.02 μmol VCE/g FW, while the bound extracts from all samples showed minimal PSC activity (Table 4). These results emphasize the antioxidant potential of fingered citron peels, particularly from the GX and ZJ varieties.

### 3.5. Inhibition of α-Glucosidase and α-Amylase

**Figure 5:**
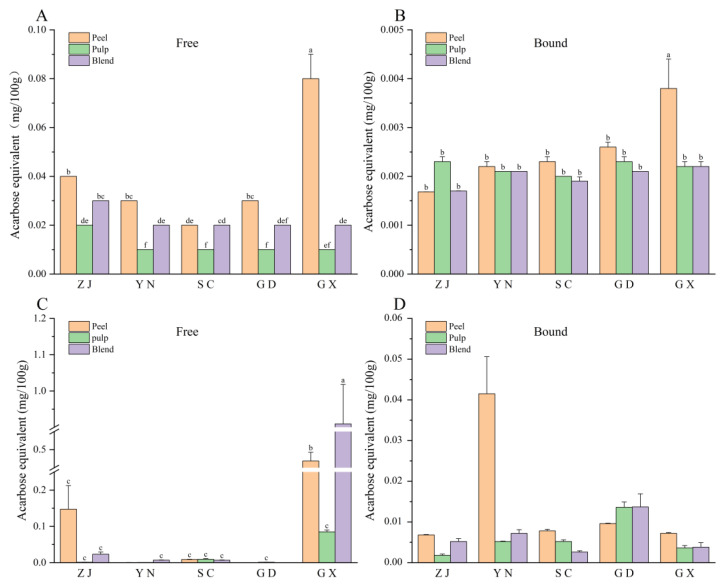
Inhibitory effects of free and bound phenolic extracts on α-glucosidase and α-amylase. (**A**) α-Glucosidase inhibition by free phenolic extracts; (**B**) α-glucosidase inhibition by bound phenolic extracts; (**C**) α-amylase inhibition by free phenolic extracts; and (**D**) α-amylase inhibition by bound phenolic extracts. Different lowercase letters indicate significant differences among groups (*p* < 0.05) according to one-way ANOVA followed by Duncan’s multiple range test. Regions as in Figure 1.

The inhibitory effects of fingered citron extracts on α-glucosidase and α-amylase showed significant differences between free and bound phenolic extracts, with free phenolic extracts exhibiting stronger enzyme inhibition. The free phenolics from GX peel exhibited the strongest α-glucosidase inhibition (76.10 ± 2.76% at 80 mg/mL), with an acarbose equivalent of 0.08 ± 0.01 mg AE/100 g (Figure 5A). The bound phenolic extracts from all five fingered citron varieties also showed some inhibitory effects on α-glucosidase, with acarbose equivalents ranging from 0.00 to 0.004 mg AE/100 g. Among these, the acarbose equivalent from GX fingered citron peel was the highest at 0.004 ± 0.001 mg AE/100 g, and the inhibition rate reached 31.78 ± 3.73% at 400 mg/mL (Figure 5B). For α-amylase, the free phenolics from the GX blend exhibited the strongest inhibition (56.77 ± 2.35% at 400 mg/mL) (Figure 5C). In comparison, the inhibition effects of bound phenolics were generally weaker, with the highest inhibition in YN peel-bound phenolics (23.05 ± 4.88%) among all bound samples (Figure 5D). These results suggest that phenolic extracts, particularly those from fingered citron peels, have great potential as natural enzyme inhibitors for postprandial blood glucose regulation.

### 3.6. Cytotoxicity and Anti-Proliferative Activity

**Figure 6:**
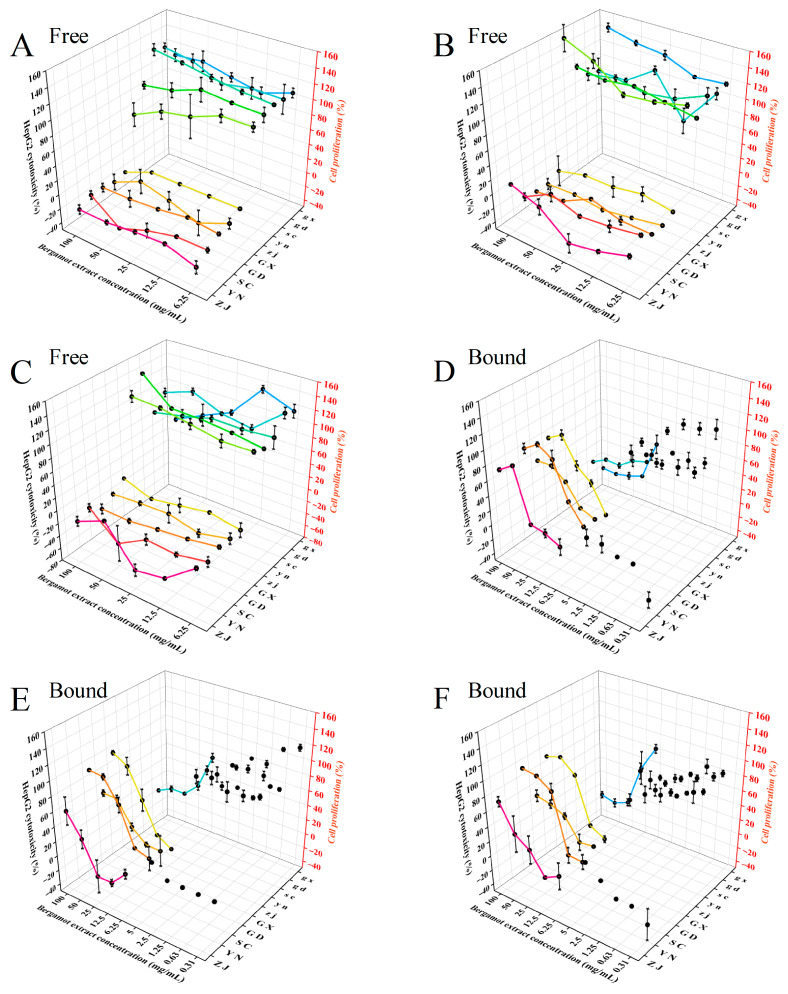
Cytotoxicity (ZJ, YN, SC, GD, GX) and anti-proliferative effects (zj, yn, sc, gd, gx) of free (**A**–**C**) and bound (**D**–**F**) phenolic extracts on HepG2 cells. (**A**,**D**: Peel; **B**,**E**: Pulp; **C**,**F**: Blend). Regions as in Figure 1.

Cytotoxicity assays revealed that free phenolic extracts were non-toxic to HepG2 cells at concentrations ≤ 10 mg/mL. Extracts from different parts of fingered citron exhibited significant anti-proliferative effects on HepG2 cells at low concentrations, with free phenolic extract from ZJ fingered citron peel showing a dose-dependent effect. The free phenolic extracts from GX blend demonstrated the highest anti-proliferative activity, inhibiting HepG2 cell growth by 54.01 ± 2.92% at 6.25 mg/mL (Figure 6). In contrast, bound phenolic extracts exhibited minimal cytotoxicity but relatively moderate anti-proliferative activity. No cytotoxicity was observed within the concentration range of 0.31–1.25 mg/mL. Among the bound phenolic extracts, those from GD fingered citron peel showed the greatest inhibitory effect on HepG2 cell proliferation (67.11 ± 1.25%) (Figure 6). Bound phenolic compounds from GX fingered citron showed no significant anti-proliferative activity, although a concentration-dependent inhibition of cell proliferation was observed at higher extract concentrations. In contrast, bound phenolic compounds from SC fingered citron demonstrated detectable anti-proliferative effects. These findings suggest that, compared to bound phenolic extract, free phenolic extract exhibited lower cytotoxicity but greater anti-proliferative effects on HepG2 cells. This difference may be attributed to variations in cellular consumption and bioavailability.

### 3.7. Glucose Consumption in IR-HepG2 Cells

**Figure 7:**
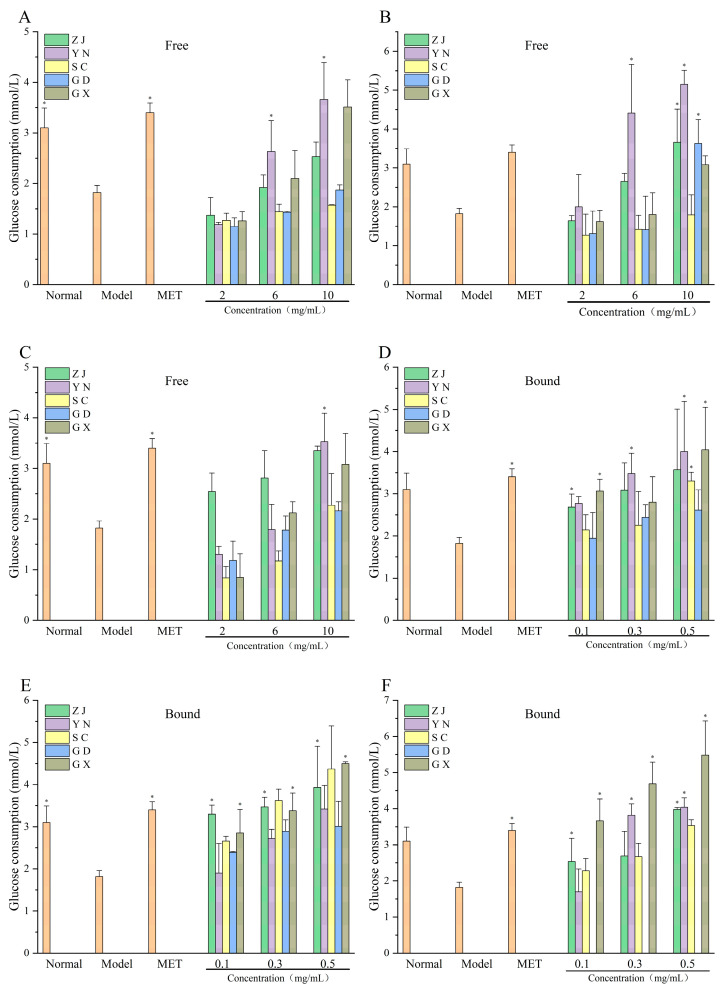
Glucose consumption in IR-HepG2 cells treated with free (**A**–**C**) and bound (**D**–**F**) phenolic extracts. Asterisks (*) indicate significant differences (*p* < 0.05). Regions as in Figure 1.

The effects of fingered citron phenolic extracts on glucose consumption in IR-HepG2 cells are shown in Figure 7. After 24 h of insulin treatment, the model group showed significantly reduced glucose consumption compared with the normal group (*p* < 0.05), indicating successful establishment of insulin resistance. Treatment with fingered citron extracts significantly improved glucose consumption relative to the model group. Among the free phenolic fractions, the high-concentration pulp extract from YN showed the highest glucose consumption (5.15 ± 0.36 mmol/L), followed by the corresponding medium-concentration group (4.41 ± 1.25 mmol/L), both of which were higher than that of the metformin group (3.40 ± 0.19 mmol/L). Among the bound phenolic fractions, the high-concentration blend extract from GX exhibited the highest glucose consumption (5.48 ± 0.98 mmol/L), followed by the high-concentration pulp extract from YN (5.15 ± 0.36 mmol/L). In contrast to the digestive enzyme inhibition assays, several bound phenolic fractions showed stronger glucose-consumption-promoting effects in the IR-HepG2 model. These results suggest that free and bound phenolic fractions may be associated with different in vitro glycemic-regulation-related endpoints.

### 3.8. Correlation Analysis

**Figure 8:**
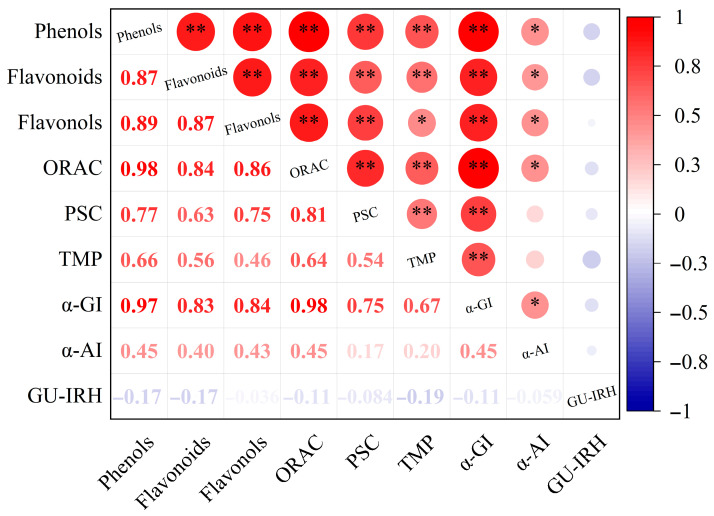
Pearson’s correlation analysis of phenolic composition and biological activities. The color scale represents the correlation coefficient (r), ranging from −1 to +1. ORAC, oxygen radical absorbance capacity; PSC, peroxyl radical scavenging capacity; TMP, types of monomeric phenols; α-GI, α-glucosidase inhibition; α-AI, α-amylase inhibition; GU-IRH, glucose consumption in insulin-resistant HepG2 cells. * *p* ≤ 0.05; ** *p* ≤ 0.01.

Figure 8 illustrates the correlation coefficient matrix between phenolic content and biological activities (ORAC, PSC, enzyme inhibition, and glucose consumption) in the studied fingered citron samples. In the present analysis, highly significant positive correlations were observed between total phenolic content and antioxidant capacity, particularly ORAC (*r* = 0.98, *p* < 0.01) and PSC (*r* = 0.77, *p* < 0.01). Furthermore, HPLC profiling revealed that hesperidin, quercetin, and 5,7-dimethoxycoumarin were among the dominant constituents in the phenolic extracts. These compounds may contribute to the observed activity patterns; however, the present correlation analysis does not allow direct attribution of α-glucosidase- or α-amylase-inhibitory activity to individual phenolic compounds. In contrast, the relationship between total phenolic content and glucose consumption in IR-HepG2 cells was less direct. Several bound phenolic fractions showed stronger glucose-consumption-promoting effects despite their lower total phenolic contents, suggesting that different phenolic fractions may be associated with different in vitro functional endpoints. However, the specific compounds and mechanisms responsible for these differences require further investigation.

## 4. Discussion

A significant positive correlation was observed between phenolic content and antioxidant-related indices, particularly ORAC (*r* = 0.98) and PSC (*r* = 0.77), in fingered citron extracts from different regions. GX peel, which contained the highest free phenolic content, exhibited the strongest radical-scavenging ability, with the highest ORAC value (30.56 ± 0.50 μmol TE/g FW), consistent with previous studies on citrus fruits [27]. This trend is also in line with the reported phytochemical characteristics of *Citrus medica*, in which flavonoids, coumarins, and phenolic acids are considered major contributors to antioxidant-related bioactivities [28]. ZJ and YN followed, whereas GD and SC showed lower ORAC values. In the PSC assay, ZJ peel (0.22 ± 0.01 μmol VCE/g FW) and YN peel (0.19 ± 0.02 μmol VCE/g FW) showed relatively higher values, while no significant differences were observed among the remaining groups. Notably, although GX peel showed the highest total phenolic content and ORAC value, ZJ peel exhibited the highest PSC value. This difference may reflect assay-specific differences in the response of individual phenolic constituents, because PSC showed a weaker correlation with total phenolic content than ORAC in the present study (*r* = 0.77 vs. *r* = 0.98). Therefore, the slightly higher PSC activity of ZJ peel may be related more to differences in phenolic composition and reaction behavior in the PSC system than to total phenolic abundance alone. In contrast, bound phenolics exhibited negligible PSC values across all regions, which may be related not only to their structural association with cell wall components, thereby limiting their release and extractability, but also to the hydrolysis-based recovery process itself. Previous studies have emphasized that bound phenolics must be released from the food matrix by alkali, acid, or enzymatic hydrolysis, and that the composition of the recovered bound fraction is therefore inherently method-dependent [29]. Although bound phenolics may possess antioxidant potential [30], the present results suggest that their release under the current extraction conditions was limited. In addition, alkaline processing can promote auto-oxidation and structure-dependent degradation of phenolic compounds, and the measured antioxidant capacity may vary depending on the assay mechanism [31]. This is particularly relevant for chlorogenic acid-related compounds, whose degradation and isomerization have been reported to accelerate as pH increases under neutral-to-alkaline conditions [32]. Therefore, the harsh alkaline hydrolysis condition used for bound phenolic extraction (4 M NaOH, 1.5 h) may have reduced the abundance of effective peroxyl-radical-scavenging constituents in the recovered bound fractions, thereby contributing to the negligible PSC values observed in the bound extracts. Because the same extraction protocol was applied uniformly to all samples in the present study, the relative comparison among bound fractions remains meaningful within this experimental framework. In the present study, ORAC and PSC were intentionally selected to evaluate antioxidant activity. This design allowed more consistent comparison of the free and bound phenolic fractions across tissues and geographical origins under the same radical system, while the fluorescence-based nature of ORAC and PSC helped reduce potential interference from extract color. Therefore, the antioxidant results reported here should be interpreted as a focused assessment of peroxyl-radical-scavenging capacity rather than a fully comprehensive antioxidant characterization. Inclusion of electron-transfer-based assays such as DPPH or FRAP would provide complementary information in future studies.

Free phenolics demonstrated significantly stronger inhibitory effects on digestive enzymes than bound phenolics. The GX free extract exhibited the highest inhibition against α-glucosidase (76.10 ± 2.76%) and α-amylase (56.77 ± 2.35%), which is in agreement with previous reports that polyphenols may help regulate postprandial glucose metabolism by inhibiting carbohydrate-hydrolyzing enzymes [33]. In contrast, bound extracts showed weaker inhibitory effects, with the highest α-glucosidase inhibition reaching only 31.78 ± 3.73% in GX peel. This lower activity may be related to the limited accessibility of bound phenolics or to differences in their chemical composition. Nevertheless, their potential sustained-release properties and in vivo metabolites deserve further investigation [34,35,36].

Cytotoxicity assays showed that free phenolic extracts were non-toxic to HepG2 cells at concentrations ≤ 10 mg/mL, while still showing anti-proliferative effects. This activity may be associated with flavonoids such as hesperidin, which has been reported to suppress cell proliferation through apoptosis-related and oxidative-stress-related pathways [37]. In contrast, bound phenolic extracts showed higher toxicity at 6.25 mg/mL, indicating that lower concentrations were more suitable for subsequent cellular assays. Accordingly, the treatment ranges used in the IR-HepG2 glucose consumption assay were set at 2–10 mg/mL for free phenolics and 0.1–0.5 mg/mL for bound phenolics.

Both free and bound extracts enhanced glucose consumption in IR-HepG2 cells; however, several bound phenolic extracts showed stronger glucose-consumption-promoting effects than the corresponding free fractions. In particular, the GX bound extract reached a glucose consumption value of 5.48 ± 0.98 mmol/L, which was numerically higher than that of metformin (3.40 ± 0.19 mmol/L). Previous studies have shown that polyphenols such as hesperidin may improve glucose metabolism in cellular models [38,39]. However, the present study did not directly examine intracellular signaling pathways. Therefore, these findings only suggest that free and bound phenolics may be associated with different in vitro glycemic-regulation-related responses, rather than demonstrating distinct mechanisms of action [40]. This discrepancy suggests that glucose-consumption-promoting activity in the IR-HepG2 model may depend more on specific phenolic composition, polarity, cellular uptake efficiency, or downstream signaling regulation than on total free phenolic abundance alone.

Pearson’s correlation analysis further supported the close relationship between phenolic indices and antioxidant capacity as well as α-glucosidase inhibition, with particularly strong correlations observed for phenolic content with ORAC (*r* = 0.98) and α-glucosidase inhibition (*r* = 0.97), whereas the correlations with α-amylase inhibition were comparatively weaker. This result suggests that phenolic compounds may share structural features contributing to both radical scavenging and digestive enzyme inhibition, including hydroxyl groups and aromatic rings involved in hydrogen bonding, electron transfer, and hydrophobic interactions [41]. By contrast, phenolic content showed only a weak negative correlation with glucose uptake in IR-HepG2 cells (*r* = −0.17), suggesting that this cellular endpoint is not determined by total phenolic abundance alone. A more plausible explanation is that glucose consumption in HepG2 cells depends more strongly on the composition of specific phenolics than on the overall free phenolic level, because structurally related flavonoids have been shown to differentially regulate glucose uptake, intracellular glucose metabolism, and glucose-transporter gene expression in HepG2 cells [42]. Moreover, because free phenolic fractions in the present study showed stronger anti-proliferative effects on HepG2 cells, part of the weak negative association may also reflect the high dependence of hepatocellular carcinoma cells on glycolytic metabolism to sustain proliferation [43].

These distinct bioactivities may be associated with differences in phytochemical composition. Previous systematic evidence on *Citrus medica* has shown that flavonoids, coumarins, and phenolic acids are among the major specialized metabolites associated with its biological activities, including antioxidant and antihyperglycemic effects. Therefore, the differences observed among the present fingered citron fractions may be associated with differences in the relative abundance and combination of these compound classes. The strong digestive enzyme inhibitory activity of GX peel may be related to its relatively high levels of quercetin (509.76 μg/g) and hesperidin (1067.13 μg/g), both of which have been reported to contribute to antioxidant activity and α-glucosidase inhibition [28,44]. Meanwhile, the stronger glucose consumption promoting effect observed for some bound phenolic fractions suggests that phenolic acids such as ferulic acid may also play a role [45]. However, the present data do not allow direct attribution of the observed activities to individual compounds. Therefore, the contributions of specific monomeric phenolics should be interpreted cautiously and require further validation using purified compounds or mechanistic experiments.

Several limitations of this study should be acknowledged. First, although some bound phenolic fractions showed stronger glucose-consumption-promoting effects, the underlying molecular mechanisms were not experimentally verified. Second, the alkaline hydrolysis condition used for bound phenolic extraction was adopted from modified literature procedures and applied uniformly across samples, but no separate optimization experiment was performed specifically for fingered citron. Therefore, degradation of alkali-sensitive compounds during extraction cannot be excluded. Third, because all quantitative results were expressed on a fresh-weight (FW) basis, potential regional differences in moisture content may also have influenced direct cross-regional comparisons. Accordingly, the present regional comparisons should be interpreted as FW-based comparative observations under the current experimental conditions.

The regional differences observed in phenolic profiles suggest that geographical origin may influence the phytochemical composition and related bioactivities of fingered citron. In the present study, GX generally showed higher phenolic, flavonoid, and flavonol levels across different fruit parts, whereas GD and YN tended to show lower values, and caffeic acid was detected only in ZJ and SC. Such variation may be associated with differences in environmental and cultivation conditions [46]. In addition, chemotype-related variation may also contribute to the observed tissue- and region-dependent patterns, because coumarin and furanocoumarin distribution in Citrus has been reported to be associated with phylogenetic background, and peel tissues generally contain a broader diversity and higher concentrations of these compounds than pulp [47]. However, because climatic and agronomic variables were not directly measured in the present study, these explanations should be regarded as possible contributing factors rather than confirmed causes. Therefore, the present regional differences should be interpreted with appropriate caution under the current FW-based experimental conditions. Across all regions, bound phenolic concentrations remained relatively low, likely due to their association with plant cell wall matrices [48]. These findings highlight the importance of considering regional variation when evaluating fingered citron as a bioactive resource and indicate that further optimization of extraction strategies may improve the utilization of bound phenolics.

## 5. Conclusions

This study revealed marked regional- and tissue-specific differences in the phenolic composition and in vitro bioactivities of fingered citron. Under the present FW-based experimental conditions, the peel, particularly that from GX, tended to show higher phenolic levels and stronger antioxidant capacity among the tested samples. Free and bound phenolic fractions exhibited different activity patterns: free phenolics were more effective in inhibiting α-glucosidase and α-amylase, whereas several bound phenolic fractions showed stronger responses in the insulin-resistant HepG2 glucose-consumption assay. These findings suggest that different phenolic fractions of fingered citron may contribute differently to distinct in vitro antioxidant- and glycemic-regulation-related endpoints. Overall, fingered citron peel represents a promising source of phenolic-rich material for further functional food research. Future studies should focus on improving the release and characterization of bound phenolics, clarifying their digestion-related transformations, and validating their bioactivities in vivo models.

## Figures and Tables

**Table 1:** Information on experimental raw materials.

Experimental Material	Production Area	Harvest Period	GPS Coordinates	Latin Species Name
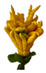	Jinhua, Zhejiang (ZJ)	October 2022	29.1068° N, 119.6442° E	*Citrus medica* L. var. *sarcodactylis* (Siebold ex Hoola van Nooten) Swingle
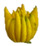	Yuxi, Yunnan (YN)	October 2022	24.3550° N, 102.5422° E	*Citrus medica* L. var. *sarcodactylis* (Siebold ex Hoola van Nooten) Swingle
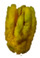	Leshan, Sichuan (SC)	September 2022	29.5520° N, 103.7660° E	*Citrus medica* L. var. *sarcodactylis* (Siebold ex Hoola van Nooten) Swingle
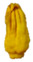	Zhaoqing, Guangdong (GD)	September 2022	23.0512° N, 112.4597° E	*Citrus medica* L. var. *sarcodactylis* (Siebold ex Hoola van Nooten) Swingle
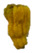	Nanning, Guangxi (GX)	October 2022	22.8167° N, 108.3167° E	*Citrus medica* L. var. *sarcodactylis* (Siebold ex Hoola van Nooten) Swingle

**Table 2:** Calibration equations and coefficients of determination (R^2^) of the 11 HPLC reference standards.

Standard	Regression Equation	R^2^
Gallic acid	y = 918.72x − 163.31	0.9921
Chlorogenic acid	y = 25,568x − 16,292	0.9931
Caffeic acid	y = 49,855x + 3865.5	0.9947
Isoscopoletin	y = 20,289x − 2879.1	0.9927
Ferulic acid	y = 57,903x − 19,253	0.997
Rutin	y = 9498.9x − 7031.4	0.9976
Hesperidin	y = 2538.4x + 15,188	0.9973
Neohesperidin	y = 4507.1x − 2708.1	0.9953
Quercetin	y = 14,613x − 32,528	0.9987
7-Hydroxycoumarin	y = 55,507x + 5525.4	0.9926
5,7-Dimethoxycoumarin	y = 37,570x − 16,319	0.9977

**Table 3:** Composition of phenolic compounds in different fingered citron extracts (μg/g).

Phenolic Compound	Zhejiang (Peel)	Zhejiang (Pulp)	Zhejiang (Blend)	Yunnan (Peel)	Yunnan (Pulp)	Yunnan (Blend)	Sichuan (Peel)	Sichuan (Pulp)	Sichuan (Blend)	Guangdong (Peel)	Guangdong (Pulp)	Guangdong (Blend)	Guangxi (Peel)	Guangxi (Pulp)	Guangxi (Blend)
Gallic Acid	26.19 ± 2.93 ^d^	9.20 ± 0.33 ^b^	10.78 ± 1.53 ^c^	58.19 ± 0.55 ^b^	14.4 ± 0.59 ^b^	12.25 ± 1.47 ^b^	23.68 ± 5.38 ^b^	11.62 ± 5.01 ^d^	5.68 ± 0.83 ^b^	27.64 ± 2.90 ^cd^	8.53 ± 1.45 ^b^	9.02 ± 0.40 ^bc^	-	7.79 ± 0.36 ^b^	15.85 ± 1.46 ^b^
Chlorogenic Acid	2.52 ± 0.11 ^d^	2.02 ± 0.19 ^b^	2.01 ± 0.09 ^c^	3.72 ± 0.90 ^b^	3.52 ± 0.10 ^b^	2.88 ± 0.49 ^b^	8.31 ± 2.41 ^b^	2.63 ± 0.66 ^d^	2.51 ± 0.33 ^b^	5.14 ± 0.06 ^cd^	1.98 ± 0.03 ^b^	2.78 ± 0.29 ^c^	7.92 ± 1.53 ^c^	2.52 ± 0.31 ^b^	2.20 ± 0.37 ^c^
Caffeic Acid	0.72 ± 0.13 ^d^	-	-	-	-	-	0.22 ± 0.34 ^b^	-	-	-	-	-	-	-	-
Isoscopoletin	2.61 ± 0.32 ^d^	1.54 ± 0.50 ^b^	1.51 ± 0.42 ^c^	6.13 ± 2.89 ^b^	0.63 ± 0.01 ^b^	2.28 ± 0.93 ^b^	0.62 ± 0.01 ^b^	10.29 ± 3.72 ^d^	5.66 ± 1.82 ^b^	9.41 ± 0.06 ^cd^	1.95 ± 0.57 ^b^	1.39 ± 0.21 ^c^	17.54 ± 2.21 ^c^	1.19 ± 0.13 ^b^	-
7-Hydroxycoumarin	1.05 ± 0.07 ^d^	1.81 ± 0.74 ^b^	2.78 ± 0.41 ^c^	1.93 ± 0.42 ^b^	-	-	1.23 ± 0.84 ^b^	2.33 ± 0.89 ^d^	1.00 ± 0.73 ^b^	0.26 ± 0.13 ^d^	0.18 ± 0.06 ^b^	-	5.93 ± 1.99 ^c^	0.09 ± 0.04 ^b^	0.06 ± 0.03 ^c^
Ferulic Acid	5.53 ± 0.42 ^d^	2.15 ± 0.19 ^b^	3.58 ± 0.47 ^c^	25.33 ± 2.79 ^b^	1.34 ± 0.31 ^b^	3.78 ± 0.64 ^b^	8.00 ± 1.61 ^b^	15.41 ± 2.54 ^d^	5.69 ± 2.81 ^b^	7.81 ± 0.08 ^cd^	1.01 ± 0.00 ^b^	1.73 ± 0.18 ^c^	19.24 ± 0.65 ^c^	-	2.79 ± 0.69 ^c^
Rutin	17.79 ± 2.32 ^d^	33.22 ± 18.36 ^b^	53.23 ± 0.85 ^b^	26.14 ± 6.85 ^b^	4.47 ± 0.08 ^b^	3.70 ± 0.86 ^b^	1.83 ± 0.93 ^b^	23.63 ± 2.07 ^d^	2.96 ± 0.06 ^b^	-	2.68 ± 0.08 ^b^	2.57 ± 0.20 ^c^	116.50 ± 7.14 ^c^	-	-
Hesperidin	397.64 ± 36.64 ^a^	6.27 ± 0.00 ^b^	19.09 ± 2.76 ^c^	473.12 ± 11.07 ^a^	139.25 ± 56.29 ^a^	88.57 ± 18.85 ^a^	20.62 ± 9.00 ^a^	351.97 ± 65.87 ^a^	92.98 ± 0.01 ^a^	104.28 ± 16.27 ^a^	61.49 ± 18.46 ^a^	86.43 ± 13.12 ^a^	1067.1 ± 37 ^a^	69.7 ± 22.63 ^a^	78.94 ± 6.44 ^a^
Neohesperidin	55.63 ± 6.86 ^c^	54.66 ± 12.89 ^a^	177.84 ± 4.51 ^a^	37.98 ± 7.95 ^b^	-	22.96 ± 14.15 ^b^	0.72 ± 0.24 ^b^	24.08 ± 6.78 ^d^	2.87 ± 0.76 ^b^	31.14 ± 0.97 ^bcd^	2.48 ± 0.08 ^b^	-	19.10 ± 6.47 ^c^	-	-
Quercetin	85.57 ± 6.54 ^b^	6.11 ± 0.12 ^b^	11.49 ± 0.90 ^c^	49.41 ± 7.68 ^b^	6.12 ± 0.32 ^b^	33.76 ± 7.09 ^b^	10.10 ± 7.30 ^b^	118.71 ± 15.68 ^b^	28.86 ± 9.61 ^b^	47.81 ± 4.89 ^ab^	9.32 ± 0.06 ^b^	15.18 ± 2.45 ^bc^	509.76 ± 33.86 ^b^	14.08 ± 1.20 ^b^	7.83 ± 0.64 ^bc^
5,7-Dimethoxycoumarin	5.01 ± 0.17 ^d^	1.09 ± 0.00 ^a^	14.35 ± 0.21 ^c^	85.62 ± 7.73 ^b^	2.07 ± 0.86 ^b^	24.84 ± 2.79 ^b^	45.97 ± 9.67 ^b^	78.94 ± 0.53 ^c^	7.22 ± 0.01 ^b^	58.15 ± 13.48 ^abc^	1.20 ± 0.00 ^a^	25.92 ± 6.21 ^b^	97.44 ± 11.96 ^c^	19.69 ± 0.66 ^b^	6.44 ± 0.44 ^bc^

Note: Mean ± SD, *n* = 3. “-” indicates below the detection limit. Values followed by different lowercase letters indicate significant differences (*p* < 0.05).

**Table 4:** PSC values of free and bound phenolic extracts from different fingered citron tissues and origins.

Origin	Extracted Fractions	Free Phenolic Compounds (μmol VCE/g FW)	Bound Phenolic Compounds (μmol VCE/g FW)
Zhejiang	Peel	0.22 ± 0.01 ^a^	-
Zhejiang	Pulp	0.11 ± 0.02 ^c^	-
Zhejiang	Blend	0.18 ± 0.02 ^b^	-
Yunnan	Peel	0.19 ± 0.02 ^b^	-
Yunnan	Pulp	-	-
Yunnan	Blend	-	-
Sichuan	Peel	0.05 ± 0.00 ^c^	-
Sichuan	Pulp	-	-
Sichuan	Blend	0.05 ± 0.01 ^c^	-
Guangdong	Peel	-	-
Guangdong	Pulp	-	-
Guangdong	Blend	-	-
Guangxi	Peel	0.17 ± 0.02 ^b^	-
Guangxi	Pulp	-	-
Guangxi	Blend	-	-

Note: “-” indicates that the PSC value is lower than the detection limit. Values followed by different lowercase letters indicate significant differences (*p* < 0.05).

## Data Availability

The original contributions presented in this study are included in the article. Further inquiries can be directed to the corresponding authors.

## Abbreviations

The following abbreviations are used in this manuscript:

FC	Flavonoid content
HPLC	High-performance liquid chromatography
IR-HepG2	Insulin-resistant HepG2
ORAC	Oxygen radical absorbance capacity
PC	Phenolic content
PSC	Peroxyl radical scavenging capacity
AE	Acarbose equivalents
CE	Catechin equivalents
GAE	Gallic acid equivalents
RE	Rutin equivalents
